# Parameters Predicting Microvascular Invasion and Poor Differentiation in Hepatocellular Carcinoma Patients with Normal Alpha-fetoprotein Level Before Liver Transplantation

**DOI:** 10.5152/tjg.2023.22538

**Published:** 2023-07-01

**Authors:** Burak M. Kılcı, Volkan İnce, Brian I. Carr, Sertaç Usta, Harika G. Bağ, Emine Şamdancı, Burak Işık, Sezai Yılmaz

**Affiliations:** 1 Department of Surgery, İnönü University, Liver Transplantation Institute, Malatya, Turkey; 2 Department of Biostatistics, İnönü University Faculty of Medicine, Malatya, Turkey; 3 Department of Pathology, İnönü University Faculty of Medicine, Malatya, Turkey

**Keywords:** Negative alpha-fetoprotein, hepatocellular cancer, microscopic venous invasion, mean platelet volume, poorly differentiation, liver transplantation

## Abstract

**Background/Aims:**

The aim of this study is to evaluate the parameters that might be associated with pathologically diagnosed microvascular invasion and poor differentiation, using complete blood count and routine clinical biochemistry test results, in hepatocellular carcinoma patients before liver transplantation.

**Materials and Methods:**

The data of patients who underwent liver transplantation for hepatocellular carcinoma at our institute, between March 2006 and November 2021, was researched retrospectively.

**Results:**

The incidence of microvascular invasion was 28.6%, poor differentiation rate was 9.3%, hepatocellular carcinoma recurrence rate after liver transplantation was 12.1%, and median time to recurrence was 13 months, in the patients with normal alpha-fetoprotein levels. After univariate and multivariate analysis, maximum tumor diameter >4.5 cm and the number of nodules (n > 5) were found to be independent risk factors for microvascular invasion, and number of nodules >4 and mean platelet volume ≤8.6 fL were found to be independent risk factors for poor differentiation. Serum alpha-fetoprotein levels were still within the normal range at the recurrence time, in 53% of the patients who had recurrence after liver transplantation, but surprisingly were elevated in 47% of the patients at time of hepatocellular carcinoma recurrence.

**Conclusion:**

In hepatocellular carcinoma patients with normal alpha-fetoprotein levels before liver transplantation, independent risk factors of the presence of microvascular invasion were maximum tumor diameter and number of nodules, and independent risk factors of poor differentiation were mean platelet volume and number of nodules. Furthermore, serum alpha-fetoprotein levels were still normal at time of recurrence in 53% of hepatocellular carcinoma patients whose alpha-fetoprotein levels were normal before liver transplantation but were elevated in 47% of the patients at recurrence time, despite having normal levels before liver transplantation.

**Main Points**

- In patients with normal blood alpha-fetoprotein (AFP) levels before liver transplantation, mean platelet volume <8.6 fL and tumor number >4 are independent risk factors for poor differentiation, while maximum tumor diameter >4.5 cm and tumor number >5 are independent risk factors for microvascular invasion.
- Clinicians may consider these findings when decision-making for treatment choice.
- Another important finding of our study is that patients with normal blood AFP levels before liver transplantation had increased blood AFP levels in 47% of patients when recurrence developed after transplantation. This finding is a unique, previously unpublished finding and clinical confirmation of intratumoral heterogeneity in hepatocellular carcinoma.

## INTRODUCTION

Hepatocellular carcinoma (HCC) is the sixth most common malignancy and the third most leading cause of cancer-associated death worldwide.^1^ The prognosis of HCC depends on several clinicopathological parameters such as high level of alpha-fetoprotein (AFP), tumor burden, degree of differentiation, presence of vascular invasion, and liver function.^2,3^ Vascular invasion is defined as the intrahepatic spread of the tumor which starts the metastatic process by tumor cells invading into the vascular endothelium. Vascular invasion is associated with more aggressive tumor behavior and is increased in patients who have a bigger tumor burden.^4,5^

Tumor differentiation is determined by cellular atypia and growth pattern, and evident pleomorphism presents in patients with poorly differentiated HCC. Poor differentiation is another sign of tumor aggressiveness, and as the tumor differentiation gets poorer, the tendency to extrahepatic metastasis risk increases. Furthermore, in poor differentiated HCC patients, the incidence of MIVI and extrahepatic recurrence increases as tumor diameter grows.^6^ Therefore, those 2 parameters are main factors that can predict patient prognosis after treatment.^7-9^ But they are not identifiable with certainty without histopathological examination before treatment.^4,5^

A high level of AFP is one of the parameters that can help to predict these histopathological parameters. However, there is still ongoing research to detect these parameters in patients with normal AFP levels. This requires high costs and complicated techniques, such as radiomics, genomics, and Positron emission tomography/computed tomography.^10,11^

The current study aims to evaluate simple, inexpensive, and reproducible parameters which can predict the presence of MIVI and poor differentiation, according to explant pathology results by using preoperative complete blood count and routine clinical biochemical test results, in HCC patients with normal preoperative AFP levels who underwent liver transplantation (LT) at the Liver Transplantation Institute, İnönü University.

## MATERIALS AND METHODS

This study has been approved by İnönü University Institutional Review Board (Approval no: 2021/2758). No informed consent was requested from patients since this is a retrospectively designed study.

### Study Population

The data of the 461 patients who underwent LT due to HCC between March 2006 and November 2021 at the Liver Transplantation Institute of İnönü University were reviewed retrospectively from a databank which was recorded prospectively and consecutively.

Demographic data of patients, the latest laboratory parameters before LT (data in the last 1 month before transplantation), body mass index, body surface area, CHILD Score, Model for End-stage Liver Disease (MELD)/Pediatric End-stage Liver Disease (PELD) scores, etiological factors for cirrhosis, transplantation type, graft-to-recipient weight ratio, and tumor characteristics according to the explant pathology report, namely maximum tumor diameter (MTD), number of nodules (NN), degree of differentiation, and presence of MIVI were recorded.

### Definition of Normal Alpha-fetoprotein Level

The normal distribution range of the AFP level in our hospital’s laboratory is between 0 and 8 ng/mL. In our study, normal AFP level was defined as ≤8 ng/mL.

### Definition of Microvascular Invasion

Microvascular invasion was defined as the detection of tumor cells in the vessel according to the explant pathology report. Microvascular invasion was defined as the presence of portal vein, hepatic artery, hepatic vein, bile duct, and non-specific angiolymphatic invasion.

### Definition of Tumor Differentiation

The degree of differentiation was defined according to the World Health Organization (WHO) grading system.^12^ The patients in the study group were examined in terms of differentiation in 2 groups as poorly differentiated and well/moderately differentiated.

### Definition of Local-Regional Treatments

Patients with a history of local-regional treatment were defined as those who had undergone any or a combination of surgical resection, radiofrequency ablation, microwave ablation, bland embolization, transarterial chemoembolisation (TACE), and transarterial radioembolisation (TARE) treatment options.

### Surgical Technique of Liver Transplantation for Hepatocellular Carcinoma at İnönü University

Our patient selection criteria and surgical method in LT for HCC have been described in our previous articles.^13-15^

### Immunosuppressive Treatment Protocol and Postoperative Follow-Up

Our immunosuppression treatment protocol and postoperative follow-up protocol after LT in patients who underwent LT for HCC were described in our previous articles.^15-17^

### Patient Inclusion and Exclusion Criteria

- Patients who had history of loco-regional treatment which can change the serum AFP level during pre-LT period (n = 76),
- Patients whose serum AFP level > 8 ng/mL (n = 236),
- Patients whose pre-Tx serum AFP level data is missed (n = 7), and
- Patients whose MIVI and degree of differentiation cannot be determined pathologically because of the autolysis of the specimen (n = 2) were excluded from this study.

Patients who had only normal serum AFP levels (n = 140) during pre-LT period were included in the study.

### Statistical Analysis

The predictive power of the laboratory parameters and tumor characteristics to detect the presence of MIVI and poor differentiation were analyzed.

In terms of the differentiation degree of the tumor, patients were divided into 2 groups as poorly and well/moderately differentiated. In terms of MIVI, the patients were again divided into 2 groups as those with and without MIVI.

Univariate and multivariate binary logistic regression analysis was performed to determine the effect of the variables on detection of poorly differentiation and MIVI positivity. Variables with a *P* value <.05 were taken into forward selection method for multivariate model to define the independent risk factors. Receiver operating characteristic (ROC) analysis was used to determine the performance of numerical variables in estimating the 2-state dependent variables and to find the cut-off values. Statistical tests were considered significant when the corresponding *P* value was less than 5%.

## RESULTS

Median age was 57.3 years and male gender was 88.6% of the study population. Demographics of the patients are summarized in Table 1.

### Incidence of Microvascular Invasion, Poorly Differentiation, and Recurrence

The incidence of the patients with normal serum AFP levels was found to be 30.3% (140/461) in our HCC study population. The rate of MIVI positivity was found to be 28.6% (n = 40), and the poor differentiation rate was 9.3% (n = 13) in these HCC patients with normal AFP level before LT. Also, in this patient group, the post-transplant HCC recurrence rate was found as 12.1% (n = 17) (Table 1). While the AFP level was still normal in 53% (n = 9) of the patients at recurrence, it was elevated in 47% (n = 8) of the patients at recurrence. Hepatic-only recurrence rate was 29.4% (n = 5), extrahepatic-only recurrence rate was 41.2 (n = 7), and both hepatic and extrahepatic recurrence rate was 29.4% (n = 5) of the recurrence patients (Table 1). The most common extrahepatic recurrence site was the lung in (n = 6).

### Relationship Between Laboratory Variables and Microvascular Invasion Positivity

In the first stage, univariate analysis was performed to evaluate the effect of the variables on the presence of MIVI. Maximum tumor diameter, NN, WBC, neutrophil count, lymphocyte count, platelet count, as well as aspartate aminotransferase, alanine aminotransferase, and c-reactive Protein (CRP) levels were found to be statistically significant in univariate analysis. The parameters that were found statistically significant in univariate analysis were involved in multivariate analysis. After the multivariate analysis of which forward selection model for variables was used, MTD and NN were detected as independent risk factors for MIVI (Table 2). Then, ROC analysis method was used to categorize MTD and NN numerically and to find cutoff values. Cutoff value was found to be 4.5 cm for MTD with 90% specificity and 62.5% sensitivity (area under curve [AUC] = 0.827, *P *< .001), and 5 for NN with 98% specificity and 40% sensitivity (AUC = 0.714, *P* = .001).

### Relationship Between Maximum Tumor Diameter, Number of Nodules, and Microvascular Invasion Positivity

Microvascular invasion positivity was also analyzed according to NN and MTD. While MIVI positivity rate was 18.1% in the patients with NN = 1, this rate rose to 88.9% in patients with NN >5, and this result was found to be statistically significant (*P* < .001). Similarly, while MIVI positivity rate was 13.2% in the patients with MTD <3 cm, this rate rose to 78.6% in patients with MTD >5 cm (*P* < .001) (Table 3).

### Relationship Between Laboratory Variables and Poor Differentiation

Later on, a univariate analysis was performed to evaluate the effect of the variables on the presence of poor tumor differentiation and NN, neutrophil count, mean platelet volume (MPV) and creatinine parameters were found statistically significant as result. Multivariate analysis with the forward-selection method was performed for these parameters. NN and MPV were detected as independent risk factors for poor differentiation (Table 4). ROC analysis was then performed to detect the cutoff values for MPV and NN. Cutoff value was found to be 8.6 fL (femto liters) for MPV with 90% specificity and 62.5% sensitivity (AUC = 0.827, *P* < .001), and was found to be 5 for NN with 98% specificity and 40% sensitivity (AUC = 0.714, *P* = .001).

### Relationship Between Mean Platelet Volume, Number of Nodules, and Poor Differentiation

To verify these results, presence of poor differentiation was analyzed according to MPV and NN. Poor differentiation rate was 4.9% in the patients whose NN = 1, and this rate rose to 33.3% in patients with NN > 5, and this result was found to be statistically significant (*P* = .002). Similarly, while the poor differentiation rate was 4.1% in patients whose MPV >8.6 fL, this rate rose to 23.1% in the patients whose MPV ≤8.6 fL (*P* < .001) (Table 5).

## DISCUSSION

Serum AFP level is the most commonly used serum parameter in the diagnosis of HCC, but it is reported in normal limits in up to 50% of HCC patients, even with advanced-stage HCC.^18^ This rate is about 41.9% in Turkey.^19^ The definition of “normal-AFP” or “negative-AFP” varies in the literature. But generally, AFP level under 20 ng/mL is accepted as “normal-AFP” or “negative-AFP.” In our study, normal-AFP patients are defined as within our clinical laboratory normal range (0–8 ng/mL). Incidence of the normal-AFP patients in this study was found to be 30.3% (140/461) and this result is similar with the literature, despite being obtained from a very specific HCC patient population, like liver transplanted.

One of the unique results of our study is that it contributes to the literature by describing the demographic and tumoral characteristics of HCC patients with normal AFP before LT. In this unique patient population, the MIVI positivity rate was 28.6%, the rate of poor HCC differentiation was 9.3%, and the recurrence rate after LT was 12.1%. At the recurrence time, 53% of the recurrent patients still had normal AFP levels, but 47% had elevated AFP levels, despite being originally in the normal range pre-Tx. These data are original and published for the first time.

Alpha-fetoprotein levels were elevated in 47% of the patients at the recurrence time and helped to diagnose the recurrence. Alpha-fetoprotein production of the tumor cells after LT which do not produce AFP before LT is a finding that supports the concept of intratumoral heterogeneity and tumor evolution in individual HCC nodules, which is mentioned in the literature before. One possible explanation of this is the proliferation of tumor clones, which produce AFP under the effect of the immunosuppressive treatment.^20^ Intratumoral heterogeneity is the presence of tumor colonies with different morphology and different degrees of differentiation in the same tumor mass. Thus, the biopsy area may not be enough to be representative of the whole tumor and its varying characteristics. Further investigations are needed concerning this topic.

Another unique finding of our study is MTD >4.5 cm and NN >5, which are independent risk factors for MIVI positivity. It has been shown in previous studies that as the tumor diameter increases, the detection of MIVI positivity and the incidence of poor differentiation also increase.^21^ Several studies show the increase of NN was related to a poorer prognosis. In many LT criteria for HCC, MTD and NN are used as patient selection criteria.^22-24^ The possible explanation for the relationship between these 2 parameters and poor prognosis is being the independent risk factor for the presence of MIVI, as we found in our study.

Another remarkable result of our study is that NN >4 and MPV ≤8.6 are independent risk factors for poor differentiation of the tumor. The relationship between the increase in the NN and poor prognosis was known previously.

Mean platelet volume is a parameter that is detected in routine complete blood count analysis and shows platelet size and activity. Studies support the idea that MPV could be a biomarker that could predict the survival rates in patients with malignancies.^25^ In this context, the low level of MPV is regarded as reflecting the presence of degranulated platelets which have secreted their tumor growth-promoting cytokines, thus being related to poor prognosis.^26^ Zang et al divided patients into 2 groups as low MPV and high MPV, according to median MPV level (11.3 fL), and figured out that the portal vein tumor thrombosis (PVTT) positivity and rates of NN >3, MTD >5 cm were higher in the low MPV level group. Low levels of MPV were found to be a statistically significant risk factor for tumor recurrence in multivariate analysis.^25^ Further studies are required regarding the significance of MPV levels in HCC patients.

The limitations of our study are that it was designed retrospectively, the number of patients was relatively small, and it included only a specific group of HCC patients who had LT.

## CONCLUSION

In conclusion, in HCC patients with normal serum AFP levels, while NN >4 is an independent risk factor for both MIVI presence and poor differentiation prediction, MTD >4.6 and MPV ≤8.6 fL are independent risk factors for MIVI positivity and poor differentiation, respectively. These parameters may be helpful for clinicians for LT decisions and the determination of prognosis in HCC patients.

Another significant result is to keep in mind that serum AFP level might be either elevated or still at normal levels in patients when the recurrence occurs after LT, despite being initially normal pre-LT.

## Figures and Tables

**Table 1.** Demographic and Clinical Variables of the Study Population

Parameters	
Age, median, year (min-max)	53.7 (8-75)
Gender, n (%)	
Female	16 (11.4)
Male	124 (88.6)
Child–Turcotte–Pugh Score, n (%)	
A	35 (25)
B	66 (47.2)
C	39 (27.8)
MELD/PELD score, n (%)	
≤14	84 (60)
>14	56 (40)
Etiology, n (%)	
Viral hepatitis	
HBV	71 (50.7)
HBV + HDV	18 (12.8)
HCV	12 (8.5)
Cryptogenic	27 (19.2)
Others	
Budd–Chiari	4 (2.8)
Ethanol	4 (2.8)
NASH	1 (0.7)
Hepatopulmonary syndrome	1 (0.7)
Fibrolamellar hepatocellular carcinoma	1 (0.7)
Autoimmune hepatitis	1 (0.7)
Transplant type, n (%)	
Living donor	133 (95)
Deceased donor	7 (5)
Milan criteria, n (%)	
Within Milan	91 (65)
Beyond Milan	49 (35)
Malatya criteria,^14^ n (%)	
Within Malatya	106 (75.7)
Beyond Malatya	34 (24.3)
Tumor differentiation, n (%)	
Well or moderate	127 (90.7)
Poor	13 (9.3)
Microvascular Invasion, n (%)	
Positive	40 (28.6)
Negative	100 (71.4)
Maximum tumor diameter (cm),	
Median (min-max)	2,5 (0.1-16)
Number of nodules, median (min-max)	1 (1-11)
Tumor recurrence, n (%)	17 (12.1)
Recurrence location, n (%)	
Hepatic	5 (29.4)
Extrahepatic	7 (41.2)
Both	5 (29.4)

HBV, Hepatitis B Virus; HCC, hepatocellular carcinoma; HCV, Hepatitis C Virus; HDV, Hepatitis Delta Virus; NASH, Non-alcoholic steatohepatitis.

**Table 2.** Univariate and Multivariate Analysis of the Variables on the Effect of MIVI Positivity

Parameters	MIVI Positivity					
	Univariate			Multivariate		
	OR	95% CI	*P*	OR	95% CI	*P*
MTD	1.657	1.366-2.009	<.001	1.492	1.230-1.810	<.001
NN	1.551	1.292-1.862	<.001	1.417	1.134-1.771	.002
WBC	1.156	1.037-1.289	.009			
Neutrophils	1.164	1.028-1.317	.016			
Lymphocytes	1.897	1.195-3.010	.007			
Platelets	1.006	1.002-1.011	.003			
AST	1.009	1.002-1.017	.015			
ALT	1.006	1.000-1.013	.044			
CRP	1.099	1.017-1.187	.017			

AST, aspartate aminotransferase; ALT, alanine aminotransferase; CRP, C-reactive protein; MIVI, microvascular invasion by tumor; MTD, maximum tumor diameter; NN, number of nodules; OR, odds ratio.

**Table 3.** MIVI Positivity Rates According to NN and MTD

NN	MIVI Positivity		*P*
	#	%	
1 (n = 82)	15/82	18.3	<.001
2-5 (n = 40)	9/40	22.5	
>5 (n = 18)	16/18	88.9	
MTD cm			
<3 (n = 76)	10/76	13.2	<.001
3-5 (n = 36)	8/36	22.2	
>5 (n = 28)	22/28	78.6	

MIVI, microvascular invasion by tumor; MTD, maximum tumor diameter, NN, number of nodules.

**Table 4.** Univariate and Multivariate Analysis of the Variables on the Effect of Poor Differentiation

Parameters	Poor Differentiation					
	Univariate			Multivariate		
	OR	95% CI	*P*	OR	95% CI	*P*
NN	1.312	1.121-1.536	.001	1.274	1.082-1500	.004
Neutrophils	1.157	1.001-1.338	.049			
MPV	0.552	0.355-.859	.008	0.579	0.363-0.923	.022
Creatinine	0.007	0.000-0.511	.023			

MPV, mean platelet volume; NN, number of nodules; OR, odds ratio.

**Table 5.** Poorly Differentiation Rates According to NN and MPV

NN	Poor Differentiation		*P*
	#	%	
1 (n = 82)	4/82	4.9	.002
2-5 (n = 40)	3/40	7.5	
>5 (n = 18)	6/18	33.3	
MPV (fL)			
>8.6 (n = 98)	4/98	4.1	.002
≤8.6 (n = 39)	9/39	23.1	

MPV, mean platelet volume; NN, number of nodules.
